# Technologies and Challenges in Proteomic Analysis of Protein *S*-acylation

**DOI:** 10.4172/jpb.1000327

**Published:** 2014-08-25

**Authors:** Bo Zhou, Mingrui An, Michael R Freeman, Wei Yang

**Affiliations:** 1 Cancer Biology Program, Samuel Oschin Comprehensive Cancer Institute, Cedars-Sinai Medical Center, Los Angeles, CA, USA; 2 Departments of Surgery and Biomedical Sciences, Cedars-Sinai Medical Center, Los Angeles, CA, USA

**Keywords:** Acylprotein thioesterase, Click chemistry, DHHC, Mass spectrometry, Palmitoyl acyltransferase, Palmitoylation, Proteomics, S-acylation, Site-specific, Substrate

## Abstract

Protein *S*-acylation (also called palmitoylation) is a pervasive post-translational modification that plays critical roles in regulating protein trafficking, localization, stability, activity, and complex formation. The past decade has witnessed tremendous advances in the study of protein *S*-acylation, largely owing to the development of novel *S*-acylproteomics technologies. In this review, we summarize current *S*-acylproteomics approaches, critically review published *S*-acylproteomics studies, and envision future directions for the burgeoning *S*-acylproteomics field. Emerging *S*-acylproteomics technologies promise to shed new light on this distinct post-translational modification and facilitate the discovery of new disease mechanisms, biomarkers, and therapeutic targets.

## Introduction

Protein *S*-acylation is the post-translational addition of long chain fatty acids to specific cysteines of proteins via labile thioester bonds [1]. Since the vast majority of *S*-acylated proteins are modified with palmitate (16:0) [2], *S*-acylation is more commonly called *S*-palmitoylation or simply palmitoylation. Nevertheless, *S*-acylation is the more accurate term because palmitoleate (16:1), stearate (18:0), oleate (18:1), arachidonate (20:4), and eicosapentaenoate (20:5) can also be incorporated onto *S*-acylated proteins [3]. As opposed to other lipid modifications such as myristoylation and prenylation, *S*-acylation is reversible. The cycling between the *S*-acylation and de-*S*-acylation states regulates protein trafficking, membrane domain partitioning, activity, stability, and complex assembly [4,5].

*S*-acylation was first reported in 1979 [6], four months earlier than the discovery of tyrosine phosphorylation [7]. It was quickly realized that *S*-acylation is important for the function of key signaling proteins, such as Ras family GTPases, Src-family tyrosine kinases, trimeric G proteins, and G-protein coupled receptors [4]. Nevertheless, after over three decades, the study of protein *S*-acylation lags far behind that of protein phosphorylation, largely due to a lack of robust methods for sensitive detection and analysis of *S*-acylation. Unlike phosphorylation and many other post-translational modifications, *S*-acylation is nonantigenic, so no anti-*S*-acylated cysteine antibody has ever been successfully produced. Traditionally, the analysis of protein *S*-acylation almost exclusively relies on [^3^H]-palmitate metabolic labeling, followed by immune precipitation and lengthy film exposure times ranging from days to weeks. Nevertheless, the past decade has witnessed tremendous advances in studying protein *S*-acylation, mostly owing to 1) the discovery of *S*-acylation and de-*S*-acylation enzymes, 2) the development of biochemical methods to selectively enrich *S*-acylated proteins and/or peptides, and 3) the rapid development of mass spectrometry (MS)-based proteomics.

Notably, recent *S*-acylproteomics (also called palmitoyl-proteomics) approaches have greatly accelerated the characterization of *S*-acylated proteins and reignited the interest in the poorly characterized dynamics of protein *S*-acylation. After almost ten years of advancement in *S*-acylproteomics, it is time to 1) summarize current proteomic approaches to the analysis of protein *S*-acylation, 2) critically review published *S*-acylproteomics studies, and 3) envision future directions for the *S*-acylproteomics field—the three major goals of this review article. For reviews on the enzymology and biological functions of *S*-acylation and the roles of *S*-acylation in physiology and pathology, see references [4,5,8,9].

## Proteomic Profiling of *S*-acylated Proteins and Sites

### Purification and identification of *S*-acylated proteins

**Figure 1:**
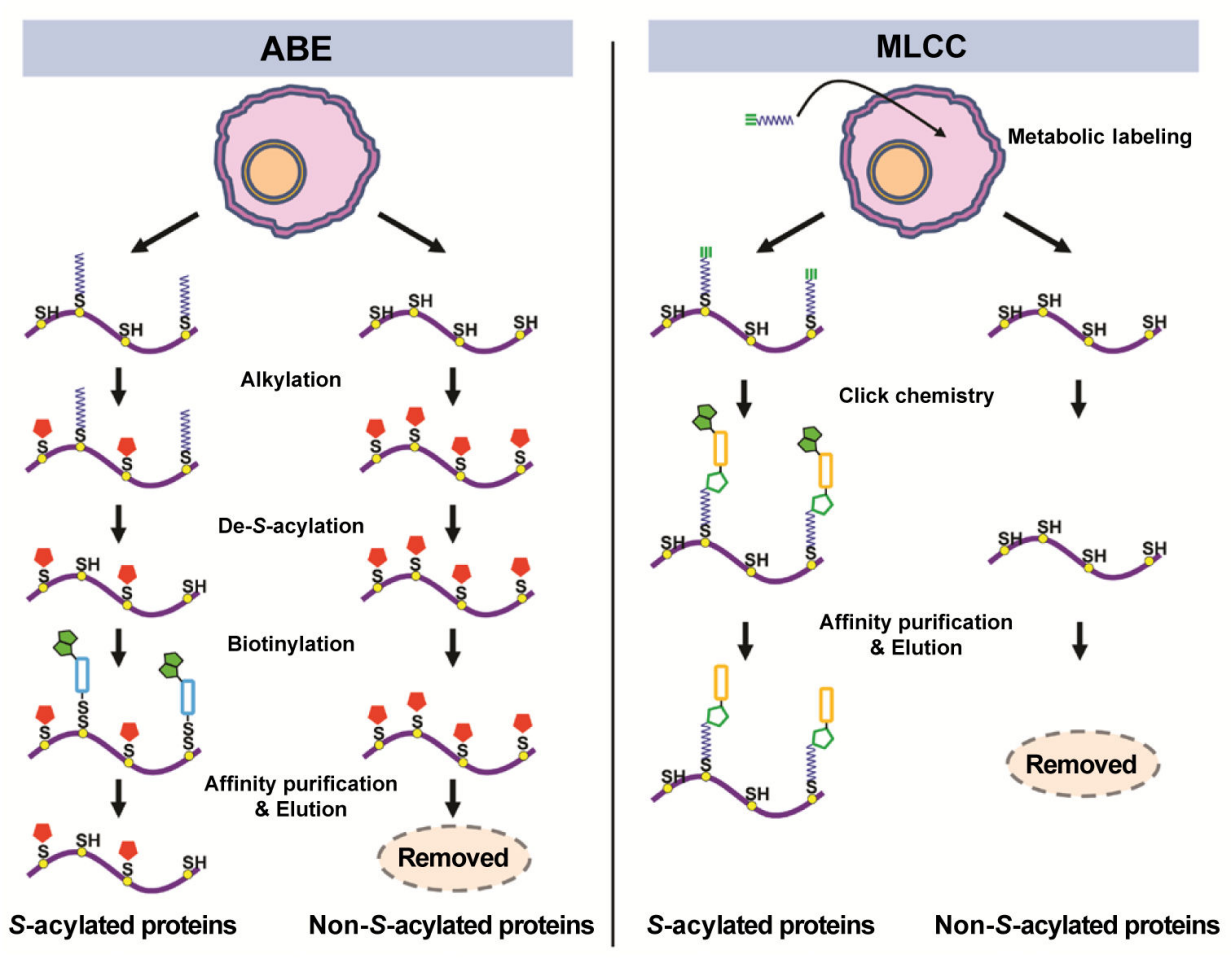
Methods for the enrichment of *S*-acylated proteins: acyl-biotinyl exchange (ABE, left panel) and metabolic labeling with a palmitic acid analog followed by click chemistry (MLCC, right panel) In ABE, free thiols are blocked by an alkylation reagent (shown as a red pentagon), such as methyl methanethiosulfonate or N-ethylmaleimide. *S*-acyl groups are specifically cleaved off by neutral hydroxylamine, and then the newly exposed cysteine residues are biotinylated by biotin-HPDP. Here, the green double pentagon shows a biotin group and the light blue square shows the linker group between the disulfide and biotin groups. The biotinylated (formerly *S*-acylated) proteins are captured by streptavidin affinity purification and eluted by a reducing agent. In MLCC, an alkyne- or azide-functionalized palmitate analog (e.g., 17-octadecynoic acid) is metabolically incorporated into native *S*-acylation sites by endogenous *S*-acylation machinery. After cell lysis, palmitate analog-labeled (*S*-acylated) proteins are conjugated to a biotin analog (e.g., azido-azo-biotin) by click chemistry, selectively enriched by streptavidin affinity purification, and eluted by sodium dithionite. In this panel, the green triple bond shows an alkyne group, the green double pentagon shows a biotin group, the green single pentagon shows a triazole group, and the orange square shows the linker group between the triazole and biotin groups.

Two methods, namely acyl-biotinyl exchange (ABE) [10] and metabolic labeling with a palmitic acid analog followed by click chemistry (MLCC) [11], have been developed to purify *S*-acylated proteins (Figure 1). In ABE, after free thiols are blocked, *S*-acylated cysteines are hydrolyzed into free cysteines by neutral hydroxylamine. The newly exposed cysteines are conjugated to a biotin analog (e.g., biotin-HPDP), so the formerly *S*-acylated proteins can be enriched by streptavidin affinity purification. In MLCC, an azide- or alkyne-functionalized (generally called bioorthogonal) palmitate analog—e.g., 14-azidotetradecanoic acid (azido-palmitate) [11], 17-octadecynoic acid (17-ODYA) [12], or 15-hexadecynyloxyacetic acid (HDYOA) [13]—is added to cell culture medium and metabolically incorporated into native *S*-acylation sites by endogenous *S*-acylation machinery. After cell lysis, labeled proteins are conjugated to a biotin analog (e.g., phosphin-biotin [11], biotin-azide [12], or azido-azo-biotin [13]) by click chemistry. The *in vitro* biotinylated (formerly *S*-acylated) proteins are then enriched by streptavidin affinity purification.

ABE and MLCC are complementary methods, so they are best used in combination for orthogonal validation. Firstly, ABE does not require metabolic labeling so it can be used to analyze *S*-acylated proteins in native cells, tissues, and biofluids. In comparison, MLCC is generally used for *in vitro* cultured cells. Moreover, to achieve best sensitivity and minimize cell death, the concentration of bioorthogonal palmitate analogs and metabolic labeling time generally need to be optimized for different cell types. Secondly, ABE has the potential to capture the full *S*-acylproteome, while MLCC only captures proteins that become *S*-acylated during the metabolic labeling window. As a result, MLCC is potentially biased towards the enrichment of *S*-acylated proteins with rapid turnover. Thirdly, ABE only provides a static profile of *S*-acylation, while MLCC can examine *S*-acylation turnover dynamics by classic pulse-chase methods. Nonetheless, it is still unknown whether and how much metabolic labeling with a bioorthogonal palmitate analog affects the dynamics of native *S*-acylated proteins. Fourthly, ABE cannot distinguish *S*-acylated proteins from thioester bond-containing non-*S*-acylated proteins, such as certain enzymes using ubiquitin, lipoic acid, or phosphopantetheine prostheses [14]. In contrast, MLCC does not enrich this group of non-*S*-acylated proteins, but it enriches for some, such as *N*- or *O*-palmitoylated proteins, metabolic enzymes associated with fatty acid synthesis and metabolism [13], and proteins labeled by the metabolic degradation products of palmitate analogs (e.g., N-myristoylated proteins [12] and lysine acetylated proteins [13,15]).

After being enriched by ABE or MLCC, *S*-acylated proteins can be profiled by MS. However, certain contaminant proteins are co-purified with *S*-acylated proteins. Thus, quantitative proteomics methods, such as spectral counting (SC) [16] and stable-isotope labeling by amino acids in cell culture (SILAC) [17], are generally used to distinguish *S*-acylated proteins (enriched in an experimental group) from contaminant proteins (equally present in both experimental and control groups). Table 1 summarizes published global profiling studies of *S*-acylated proteins. It should be noted that, in these studies, statistical tests used to distinguish *S*-acylated proteins from contaminant proteins were performed at various levels, ranging from rigorous tests to no statistical analysis at all. In early *S*-acylproteome profiling studies, SC was used to discriminate *S*-acylated proteins from co-purified contaminant proteins. However, although easy to implement, SC is only semi-quantitative and not very reliable for the quantitation of low-abundance proteins or modest changes. Recently, more accurate quantitative proteomics methods, particularly SILAC, were used to distinguish *S*-acylated proteins from contaminant proteins. Due to the high accuracy of SILAC, even small (~1.5-fold) enrichment can be reliably detected, thus more candidate *S*-acylated proteins can be identified than using SC.

These *S*-acylproteomics profiling studies (summarized in Table 1) lead to the annotation of an unexpected large number of *S*-acylated proteins, indicating that *S*-acylation is a pervasive modification and important for a variety of functions. To increase the sensitivity and speed of detecting *S*-acylated proteins, the current methods need to be further improved. For ABE, one issue is that the whole procedure is laborious and time-consuming, largely due to the multiple steps of precipitation and resolubilization of samples. The acyl-RAC (resin-assisted capture) [18] and acyl-TPC (thiopropyl captivation) [19] methods, same protocol with different names, use thiolpropyl sepharose to eliminate the biotin enrichment steps so the purification scheme is simplified. Another key issue of ABE is that incomplete alkylation of free thiols would lead to major background in the subsequent biotinylation step. Our survey of the published ABE-based *S*-acylproteome profiling studies suggested that contaminant proteins may account for about 10–40% of total purified proteins in amount. Given that contaminant proteins will mask the MS identification of *S*-acylated proteins, especially those of low abundance, the alkylation step needs to be optimized to reduce the overall background, so the sensitivity of detecting low-abundance *S*-acylated proteins can be increased.

### Purification of *S*-acylated peptides and identification of *S*-acylation sites

*S*-acylation is commonly observed on cysteines proximal to protein N- or C-termini, within cysteine-rich motifs, or close to the cytosolic side of transmembrane domains [4]. Nevertheless, no unique consensus sites for *S*-acylation have been definitely established yet. Although some algorithms have been developed to predict *S*-acylation sites [37], experimental determination of *S*-acylation sites has to be performed. So far, only three proteomics studies have been conducted to profile *S*-acylation sites on a relatively large scale (Table 2).

Zhang et al. [36] developed a method called palmitoyl-cysteine isolation capture and analysis (PICA) to identify *S*-acylation sites. In PICA, after using MMTS to block free cysteines, acyl groups are removed from *S*-acylated cysteines with neutral hydroxylamine, and the newly exposed (formerly *S*-acylated) cysteines are labeled with thiol-reactive cleavable isotope-coded affinity tag (cICAT) reagents [38]. Proteins are digested into peptides, and then cICAT-labeled (formerly *S*-acylated) peptides are enriched by avidin purification and acid-cleaved to remove biotin groups. In this study, 50 cICAT-labeled peptides were enriched from human cervical cancer HeLa cells and identified with *p*<0.05. Nevertheless, although the authors showed several pieces of evidence that PICA can enrich *S*-acylated proteins with high selectivity, they did not directly analyze whether the identified peptides are hydroxylamine sensitive, so it remains unclear whether certain identified peptides are contaminant peptides.

We developed another method called Palmitoyl Protein Identification and Site Characterization (PalmPISC) and reported so far the largest dataset of *S*-acylation sites [21]. In PalmPISC, disulfide bonds are broken by TCEP, a reducing agent that does not cleave thioester bonds, followed by the blockage of all free thiols by N-ethylmaleimide. Then *S*-acyl groups on cysteine residues are replaced by biotinyl groups via ABE. After *in vitro* biotinylated (formerly *S*-acylated) proteins are digested into peptides, biotinylated (formerly *S*-acylated) peptides are enriched by streptavidin affinity purification, eluted by TCEP, and identified by liquid chromatography-tandem mass spectrometry. A total of 527 cysteine-containing peptides were identified. Using SC quantitation, we identified 127 candidate *S*-acylation sites with high confidence and 39 sites with medium confidence. Interestingly, our site-specific analysis showed that several DHHC-PATs are *S*-acylated on multiple cysteine residues.

Forrester et al. [18] developed yet another method called *S*-acylation by resin-assisted capture (acyl-RAC), in which thiolpropyl sepharose is used to replace the biotin enrichment steps, so *S*-acylated peptides can be more rapidly purified. The authors coupled acyl-RAC with isobaric tagging for relative and absolute quantitation (iTRAQ) [39] to distinguish hydroxylamine-sensitive peptides (candidate *S*-acylated peptides) from hydroxylamine-resistant peptides (contaminant peptides). A total of 84 cysteine-containing peptides were identified. Surprisingly, no iTRAQ ratios were provided for the identified peptides, so it is unclear whether some cysteine-containing peptides are contaminant peptides.

Interestingly, all these site-specific studies employed ABE-derived methods. Theoretically, the MLCC methods can also be used to enrich *S*-acylated peptides. Nevertheless, they introduce a bulky (400–900 Da) group that is very hydrophobic to each *S*-acylation site. Consequently, the enriched *S*-acylated peptides, especially those that are also myristoylated or prenylated and those that are dually or multiply *S*-acylated, can easily get lost during sample preparation, transfer, and storage. In addition, the high hydrophobicity of the enriched peptides poises a great challenge for separation on the C18 reversed-phase columns. Compared with uncleavable biotin-azide [12], cleavable azido-azo-biotin allows the removal of hydrophobic biotin groups with sodium dithionite [22], thus reducing the size and hydrophobicity of MLCC-introduced tags. Nevertheless, substantial progress has to be made to address the challenges.

With the development of increasingly sensitive proteomics instrumentations and methods, we believe much more comprehensive characterization of *S*-acylation sites, at least by using the ABE methods, will be reported in the near future. The combination of different endoproteases for protein digestion will surely provide a more complete coverage of *S*-acylation sites.

### Proteomic Profiling of Substrates of a (de-)*S*-acylation Enzyme

*S*-acylation can be spontaneous [40], but it is more generally catalyzed by a group of Asp-His-His-Cys (DHHC) motif-containing palmitoyl acyltransferases (PATs) encoded by *ZDHHC* genes (7 in yeast, 23 in human, 24 in mouse and *Arabidopsis*) [8]. Each PAT has four to six transmembrane domains and a cysteine-rich domain harboring the catalytic DHHC motif. In addition, some DHHC-PATs have putative domains involved in protein-protein interactions and substrate recognition (e.g., C-terminal PDZ binding motifs and N-terminal ankyrin repeats). In cells, DHHC-PATs are localized to different compartments such as the endoplasmic reticulum, the Golgi apparatus, plasma membrane, and endocytic vesicles. *S*-acylated proteins may be de-*S*-acylated by a small family of enzymes called acylprotein thioesterases (APTs) [9], but it remains enigmatic whether other de-*S*-acylation enzymes exist [41]. Accumulating evidence suggests that mutations and deregulation of *ZDHHCs* and *APTs* are associated with many diseases such as cancer, Huntington’s disease, Alzheimer’s disease, schizophrenia, bipolar disorder, X-linked mental retardation, and osteoporosis [37], suggesting that the DHHC-PATs and APTs are potentially novel therapeutic targets. To understand the functions of disease-associated PATs/APTs, it is critical to link individual enzymes to their corresponding substrates. To date, most PAT-substrate and APT-substrate pairs are established on a small scale (e.g., by ABE/MLCC followed by western blotting) [42]. The powerful *S*-acylproteomics technologies, coupled with genetic manipulation of individual DHHC-PATs and APTs, have great potential to identify novel substrates with high speed and sensitivity. To our knowledge, no large-scale analysis of de-*S*-acylation substrates of any specific APTs has been reported, although substrates of a broad spectrum of thioesters have been identified [27]. Nonetheless, at least six *S*-acylproteomics studies have already been conducted to identify substrates of certain DHHC-PATs—two in yeast, one in plant, and three in mammals (Table 3).

To map the substrates for the seven DHHC-PATs in *Saccharomyces cerevisiae*, Roth et al. [14] compared the differences in the *S*-acylation levels of 30 abundant *S*-acylated proteins before and after the deletion of one to six PATs. They found that DHHC-PATs have both discrete and overlapping specificities. In another yeast study, Zhang et al. [33] found that global *S*-acylation is changed during meiosis in the fission yeast *Schizosaccharomyces pombe* and that the Erf2-Erf4 complex drives major *S*-acylproteome changes. To identify Erf2 substrates that are selectively *S*-acylated during meiosis, the authors coupled 17-ODYA-based MLCC with SC and identified 238 candidate substrates. They validated that Isp3 and Rho3 are Erf2 substrates and that Rho3 *S*-acylation is important for Erf2-induced meiosis.

To identify the substrate of Tip Growth Defective 1 (TIP1), a plant DHHC-PAT, Hemsley et al. [32] combined ABE with iTRAQ to compare the *S*-acylproteome differences between *Arabidopsis* callus cells expressing *tip1-WT* or *tip1-2* mutant. 103 proteins were identified with at least 1.5-fold under-representation in the *tip1-2* samples, suggestive of TIP1 substrates. Nevertheless, whether the under-representation is caused by reduced protein abundance or by decreased *S*-acylation levels is unknown. Furthermore, no candidate TIP1 substrate was verified.

In addition to the studies in yeast and plants, three *S*-acylproteomics studies were conducted to identify the substrates of DHHC-PATs in mammals. In the first study, to identify human DHHC2 substrates, Zhang et al. [33] integrated *ZDHHC2* siRNA knockdown, PICA, and cICAT quantitation to identify cysteine residues *S*-acylated by DHHC2 [36]. Among a total of 50 identified peptides, only one *S*-acylated peptide derived from cytoskeleton-associated protein 4 (CKAP4) was significantly (*p*<0.05) downregulated after DHHC2 knockdown. Subsequent functional assays confirmed that CKAP4 is a substrate of DHHC2. In the second study, to profile mouse DHHC5 substrates, Li et al. [28] coupled 17-ODYA-based MLCC with SILAC to compare the *S*-acylproteome differences between neuronal stem cells cultured from forebrains of normal or DHHC5-GT (gene trapped) mice. About 20 *S*-acylated proteins were identified to be at least 2-fold downregulated in DHHC5-GT cells, suggestive of DHHC5 substrates. However, subsequent proteomic analysis of total membrane lysates revealed that the *S*-acylation levels of at least ten candidate DHHC5 substrates were unchanged—the downregulation of these candidate substrates actually arised from decreased protein abundance. A potential explanation is that DHHC5-GT did reduce the *S*-acylation levels of the candidate substrates, but likely due to a tight connection between *S*-acylation and protein stability, reduced *S*-acylation levels resulted in decreased protein abundance. Further analysis of flotillin-2, a selected candidate, confirmed it is a DHHC5 substrate. Nevertheless, it remains to be determined whether other candidates, especially those whose *S*-acylation levels were unchanged by DHHC5-GT, are also substrates for DHHC5. In the third study, to identify the substrates of DHHC17 (also called Huntingtin-interacting protein 14, HIP14), a PAT important for Huntingtin’s disease, Wan et al. [43] coupled ABE with stable isotope labeling in mammals (SILAM) to analyze brain *S*-acylproteome difference in *Hip14-WT* and *Hip14-gt* mouse [34]. From ~300 candidate *S*-acylated proteins, 17 are significantly (*p*<0.05) changed with 10–36% reduction, suggestive of HIP14 substrates. Nevertheless, subsequent western blotting analysis of three significantly changed proteins (flotillin-1, flotillin-2, and glutamine synthase) indicated that their abundance changes are well correlated with the *S*-acylated protein level changes. Moreover, overexpressing HIP14 in yeast does not increase the *S*-acylation of flotillin-1 or -2. Therefore, at least some of the identified candidate substrates are only false positives.

**Figure 2:**
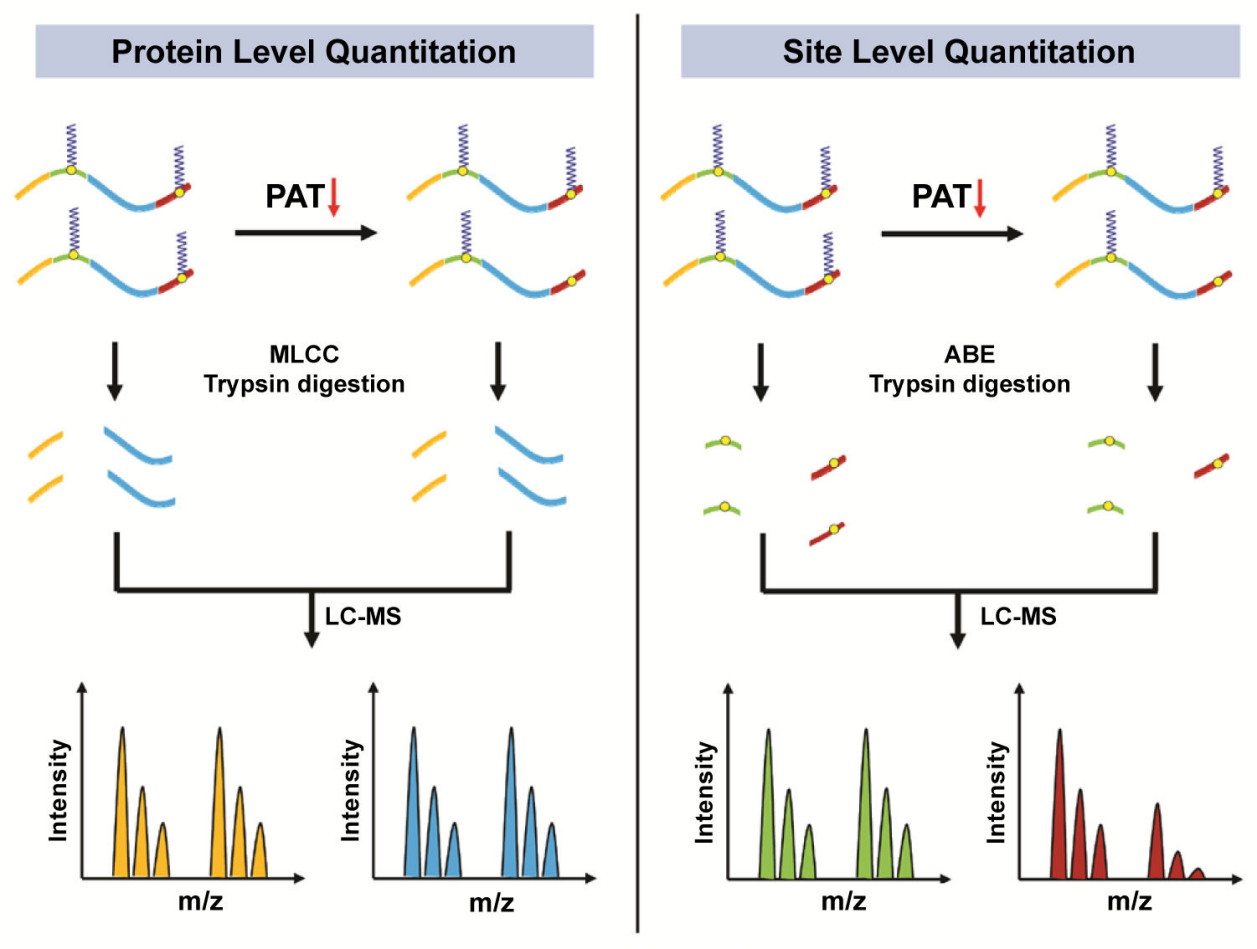
Studying *S*-acylation changes at the protein level has its inherent limitations for proteins with two or more *S*-acylation sites The mutation or reduced expression of a DHHC-PAT results in decreased *S*-acylation level of certain substrate *S*-acylation sites. In the currently dominant strategy (left panel), *S*-acylated proteins are purified using the MLCC method and digested into tryptic peptides for LC-MS analysis. However, for a dually or multiply *S*-acylated protein, if the *S*-acylation level for one site is unchanged, then the same amount of *S*-acylated proteins will be purified before and after DHHC-PAT loss. Because with the MLCC method almost all *S*-acylated peptides get lost, it wrongly appears that the *S*-acylated protein is unchanged and thus not the substrate for the DHHC-PAT. In comparison, the site-specific analysis strategy is more appropriate to determine whether a dually or multiply *S*-acylated protein is a DHHC-PAT substrate (right panel). This strategy can easily distinguish substrate *S*-acylation sites from non-substrate *S*-acylation sites on the same protein.

In summary, the identification of substrates for DHHC-PATs in mammals (and potentially in plants) using quantitative *S*-acylproteomics technologies has not been a great success. Novel approaches need to be developed for global profiling of PAT substrates. For instance, if it is a widespread phenomenon that reduced protein *S*-acylation causes protein degradation (and thus reduced protein abundance), more rapid DHHC-PAT ablation (e.g., by chemical inhibition) and/or pharmacologic inhibition of protein degradation may help to untangle the intertwined relationship between protein *S*-acylation and stability. In addition, it should be noted that for dually or multiply *S*-acylated proteins, the *S*-acylation level changes caused by DHHC-PAT gene mutation or knockdown, when measured at the protein level, only reflects the changes of one site that has the least *S*-acylation level reduction (Figure 2). In this case, direct analysis of *S*-acylated proteins will underestimate the true number of DHHC-PAT substrates. In contrast, site-specific analysis will yield more meaningful results and thus should be adopted.

### Proteomic Profiling of off-targets of a (de-)*S*-acylation Inhibitor

Due to the importance of protein *S*-acylation in diseases and the perspective that certain DHHC-PATs and APTs may serve as key therapeutic targets, the development of pharmacological inhibitors of PATs and APTs has garnered increasing interest. Currently, several small molecules such as cerulenin, tunicamycin, 2-bromopalmitate, and Compound V are used as PAT inhibitors, and potent inhibitors such as palmostatin B, palmostatin M, inhibitor 21, and inhibitor 1 have recently been developed to inhibit APTs [37,44].

These enzyme-specific inhibitors will undoubtedly accelerate the study of dynamic *S*-acylation. However, in addition to inhibiting their main targets, inhibitors may bind and inhibit off-targets, causing side effects. Thus, the knowledge of potential off-target effects of an inhibitor is important not only for understanding its mode of action but also for further improvement of the inhibitor. By coupling click chemistry with proteomics, off-targets can be rapidly profiled. To identify the potential targets of 2-bromopalmitate (2BP), a general *S*-acylation inhibitor with controversy, Davda et al. [45] synthesized the ω-azido analog of 2BP (2BPN3) and applied it to purify 2BP targets. Label-free quantitative proteomics analysis identified 450 proteins that bind to 2BPN3, with an at least 5-fold enrichment as compared to the control. Although the candidate targets include at least five DHHC PATs, they also contain some *S*-acylated proteins and many non-*S*-acylated proteins. This indicates that 2BP is a non-selective probe with many targets beyond DHHC PATs. Thus, it should not be used as direct proof of enzymatic *S*-acylation.

With an increasing number of potent and specific PAT and APT inhibitors becomes available, our understanding of the dynamics of *S*-acylation will be greatly enhanced. We envision that more proteomic profiling studies of off-targets, especially for the inhibitors that may enter preclinical or clinical trials, will be conducted to help understand the potential side effects and toxicity of the drugs and guide the improvement of the inhibitors.

### Proteomic analysis of dynamic *S*-acylation

Akin to protein phosphorylation, protein *S*-acylation is a dynamic and reversible process. Understanding how *S*-acylated proteins are globally regulated is essential to determine the roles of this important and pervasive modification. Since late 2011, five proteomic studies of dynamic *S*-acylation have been reported (Table 4).

Martin et al. [27] described the first proteomic analysis of dynamic *S*-acylation. To identify proteins that rapidly cycle their *S*-acylation state, the authors performed a MLCC-based pulse-chase study. Using SILAC quantitation, the authors identified about 80 *S*-acylated proteins as candidates with rapid *S*-acylation cycling. Using the same strategy, the authors also surveyed for which proteins, their *S*-acylation cycling is enzymatically regulated [27]. They found that the *S*-acylation cycling of 49 (out of 328) proteins was blocked by hexadecylfluorophosphonate, which inhibits many serine hydrolases including APT1 and APT2.

Jones et al. [31] coupled ABE with stable isotope dimethyl labeling to identify *S*-acylated proteins regulated by 2BP. They found that 6 h 2BP treatment results in >21.8% decrease in *S*-acylated protein levels for 67 proteins in parasite *Plasmodium falciparum*.

Wan et al. [34] compared the *S*-acylproteome differences in WT and YAC128 (a mouse model of Huntingtin’s disease) mouse brains. 19 proteins showed significant (*p*<0.05) changes, with a level difference of −42% to +33%. Nevertheless, western blotting analysis of two significantly changed proteins (carbonic anhydrase 2 and glutamine synthase) demonstrated that their *S*-acylprotein level changes are more likely caused by reduced protein abundance than by decreased *S*-acylation.

Wei et al. [35] coupled ABE with SILAC to investigate the insulin-regulated *S*-acylproteome in human umbilical vein endothelial cells. 26 proteins were identified to be significantly (*p*<0.05) regulated, with a ratio of above 2 or less than −3.

In short, in these published studies, only binary comparisons were performed. Given the dynamics of *S*-acylation, we envision that the spatiotemporal changes of *S*-acylated proteins will be investigated by combining ABE/MLCC with emerging multiplexed quantitative proteomics methods. The increase in proteomic throughput will greatly accelerate our understanding of dynamic *S*-acylation cycling *in vitro* and *in vivo*.

## Important Challenges in the *S*-acylproteomics Field

In addition to the *S*-acylproteomics studies summarized above, some important aspects of *S*-acylation have not been investigated using proteomics methods. Below we briefly describe selected challenges for the *S*-acylproteomics field.

### Direct analysis of native *S-*acylated peptides

*S*-acylated proteins are modified by a heterogeneous population of long chain fatty acids. Though palmitate is the predominant form, other fatty acids such as palmitoleate, stearate, oleate, arachidonate, and eicosapentaenoate can also modify proteins on cysteine residues [3] and may target *S*-acylated proteins to different membrane domains. Unfortunately, both ABE and MLCC ignore the native *S*-acyl chain attachment. To determine the fatty acids attached to a specific *S*-acylation site, MS analysis of intact *S*-acylated peptides can provide direct evidence. It has been shown that at least singly or dually *S*-palmitoylated peptides can be separated by C18 reversed-phase liquid chromatography and sequenced by MS [46]. Thus, the real challenges are how to keep thioester bonds intact during sample preparation and ionization, how to separate native *S*-acylated peptides from non-*S*-acylated peptides, and how to keep highly hydrophobic *S*-acylated peptides in solution.

### Proteomic analysis of *S*-acylation site stoichiometry

Many *S*-acylated proteins may represent only fractional site occupancy. To date, no global analysis of *S*-acylation site stoichiometry has been reported, though a small scale analysis of *S*-acylation stoichiometry using acyl-RAC and western blotting has recently been published [34]. Multiplexed targeted MS or directed MS may play a role in addressing this challenge.

### Cross-talk with other modifications

Cysteine residues can not only be acylated but also be oxidized, nitrosylated, or glutathionylated. These cysteine-specific modifications may be competitive in regulating protein localization and activity. In addition, several studies showed that *S*-acylation prevents protein ubiquitination and degradation. It would be interesting to determine whether this is a widespread phenomenon, as suspected in two aforementioned PAT-substrate studies [28,34]. Additionally, global *S*-acylproteome profiling studies demonstrated that certain kinases and phosphatases can be *S*-acylated. The cross-talk between *S*-acylation and phosphorylation may play an important role in regulating signal transduction and disease progression. Here, the major challenge for proteome-scale analysis of modification cross-talk is that only a tiny fraction of proteins are modified by both *S*-acylation and another type of modification, thus more sensitive methods have to be developed.

## Summary

In the past decade, the study of protein *S*-acylation is greatly accelerated by the development of ABE and MLCC methods as well as their derivatives for the purification of *S*-acylated proteins or peptides. Quantitative proteomics analysis of purified proteins have identified thousands of putative *S*-acylated proteins in total, suggesting that *S*-acylation is a pervasive modification and important for various cellular functions. Global analyses of purified *S*-acylated peptides have identified ~200 candidate *S*-acylation sites. More comprehensive localization of *S*-acylation sites waits to be performed. The studies to establish the global linkage between an individual PAT/APT enzyme and its substrates are not very successful, so novel approaches need to be developed to map the substrates of a PAT/APT. The combination of click chemistry with quantitative proteomics is a powerful approach to identify off-targets of PAT/APT inhibitors. The dynamics of protein *S*-acylation has already been investigated by coupling ABE/MLCC with duplex quantitative proteomics technologies. The study of dynamic *S*-acylation will be accelerated when emerging multiplexed quantitative proteomics are adopted. In addition, proteome-scale analysis of intact *S*-acylated peptides, *S*-acylation site occupancy, and cross-talk between *S*-acylation with other modifications remain unsolved challenges in the *S*-acylproteomics field. In short, the study of protein *S*-acylation has been revolutionized by burgeoning *S*-acylproteomics technologies. Further *S*-acylproteomics studies hold great potential of revealing unknown functions and mechanisms of protein *S*-acylation as well as discovering novel disease mechanisms, biomarkers, and therapeutic targets.

## Figures and Tables

**Table 1:** Summary of global profiling studies of *S*-acylated proteins.

Epub Date	Source	Enrichment Method	Identified Proteins	Identification Confidence	Quantitation Method	EXP/CON cutoff	Candidate *S*-acylproteins	Reference
06/02/2006	Yeast *Saccharomyces cerevisiae*	ABE	1,557	≥ two peptides (XCorr ≥ 1.8 (+1), 2.5 (+2), and 3.5 (+3), and ΔCn ≥ 0.08)	SC	5.5	70	[14]
10/30/2007	Rat liver mitochondria	MLCC (azidopalmitate)	21	Peptide mass fingerprinting (*p*<0.05)	Gel band intensity	n/a	21	[11]
12/18/2008	Rat embryonic cortical neurons and synaptosomes	ABE	1,647 (neurons); 1,337 (synaptosomes)	≥ two peptides (XCorr ≥ 1.8 (+1), 2.5 (+2), and 3.5 (+3), and ΔCn ≥ 0.08)	SC	Graphic analysis	163 (high)a) 332 (medium)b)	[20]
01/11/2009	Human Jurkat T cells	MLCC (17-ODYA)	n/a	≥ two peptides (XCorr ≥ 1.8 (+1), 2.5 (+2), and 3.5 (+3), and ΔCn ≥ 0.08)	SC	5 (high) 2–5 (medium)	125 (high) 199 (medium)	[12]
10/02/2009	Human prostate cancer DU145 cells	ABE (PalmPISC)	928	≥ two different peptides (*p*<0.05)	SC	6.7 (high) 3.0–6.7 (medium)	169 (high) 224 (medium)	[21]
07/04/2010	Mouse dendritic DC2.4 cells	MLCC (17-ODYA)	n/a	n/a	SC	10 (high) 5–10 (medium)	60 (high) 97 (medium)	[22]
11/14/2010	Human Jurkat T cells	MLCC (five az/alk fatty acid analogs)	n/a	≥ two peptides (*p*<0.05)	SC	5 (high) 3–5 (medium)	178 (high) 194 (medium)	[23]
12/30/2010	Parasite *Trypanosoma brucei*	ABE	1,672	FDR=0.016	SC	2 and QSpec FDR=0.01	124	[24]
04/03/2011	Human cervical cancer HeLa cells	MLCC (HDYOA)	53	n/a	SC	n/a	n/a	[13]
07/23/2011	Mouse RAW 264.7 macrophages	ABE	1,183	FDR<0.01 (≥ two unique peptides)	SC	n/a	80 (FDR=0.05); 21 (FDR=0.10–0.05)	[25]
08/02/2011	Human platelets	ABE (PalmPISC)	1,300	FDR<0.01	SC	3	215	[26]
11/06/2011	Mouse T-cell hybridoma cells derived from BW5147	MLCC (17-ODYA)	n/a	FDR<0.01	SILAC2plex	1.5	415 (PAc) control) 338 (Hydd) control) 338 (both)	[27]
11/11/2011	Mouse neural stem cells	MLCC (17-ODYA)	n/a	Peptide FDR<0.01	SILAC2plex	5	434 (PA control) 940 (Hyd control) ~300 (both)	[28]
04/10/2012	Human endothelial EA.hy926 cells	ABE	n/a	n/a	No	No	154	[29]
05/17/2012	Human B lymphoid cells	ABE	493	FDR<0.01	SC	Spectral index>0.54	139	[30]
08/16/2012	Parasite *Plasmodium falciparum*	ABE and MLCC (17-ODYA)	1,752	FDR<0.01	SILAC2plex	n/a	353 (ABE), 176 (MLCC)	[31]
12/17/2012	Arabidopsis root-derived callus cells	ABE	924	FDR<0.01	iTRAQ4plex	1	144 (high) 115 (medium)	[32]
01/01/2013	Mouse epididymal fat pads and 3T3-L1 adipocytes	ABE (Acyl-TPC)	856	≥ three unique peptides	No	No	856	[19]
07/02/2013	Yeast *Schizosaccharomyces pombe*	MLCC (17-ODYA)	n/a	n/a	SC	2	n/a (>238)	[33]
11/07/2013	Mouse brain	ABE	~300	FDR<0.02 (≥ 40 peptides)	No	No	~300	[34]
12/19/2013	Human umbilical vein endothelial cells	ABE	~1,700	n/a	SILAC2plex	1.5	~500	[35]

a) High-confidence;

b) Medium-confidence;

c) Palmitate;

d) Hydroxylamine.

**Table 2:** Summary of proteomic analysis of *S*-acylation sites.

Epub Date	Source	Enrichment Method	Identified Cys- containing Peptides	Identification Confidence	Quantitation Method	EXP/CON Cutoff	Candidate Palmitoylation Site	Reference
02/22/2008	Human cervical cancer HeLa cells	ABE (PICA)	50	*p*<0.05	No	n/a	57	[36]
10/02/2009	Human prostate cancer DU145 cells	ABE (PalmPISC)	527	*p*<0.05	SC	2.4 (high)a) 2/0 (medium)b)	127 (high) 39 (medium)	[21]
11/02/2010	Human embryonic kidney HEK-293 cells	ABE (acyl-RAC)	84	FDR=0.012	iTRAQ2plex	n/a	93	[18]

a) High-confidence;

b) Medium-confidence.

**Table 3:** Summary of proteomics studies to identify DHHC-PAT substrates.

Epub Date	Source	Enrichment Method	Quantitation Method	Enzyme	Reference
06/02/2006	Yeast *Saccharomyces cerevisiae*	ABE	SC	Akr1, Akr2, Erf2-Shr5, Swf1, Pfa3, Pfa4, Pfa5	[14]
02/22/2008	Human cervical cancer HeLa cells	ABE (PICA)	cICAT	DHHC2	[36]
11/11/2011	Mouse neural stem cells	MLCC (17-ODYA)	SILAC2plex	DHHC5	[28]
12/17/2012	*Arabidopsis* root-derived callus cells	ABE	iTRAQ	TIP1	[32]
07/02/2013	Yeast *Schizosaccharomyces pombe*	MLCC (17-ODYA)	SC	Erf2	[33]
11/07/2013	Mouse brain	ABE	SILAM	DHHC17 (HIP14)	[34]

**Table 4:** Summary of proteomic studies of dynamic *S*-acylation.

Epub Date	Source	Treatment	Enrichment Method	Quantitation Method	Quantitation Cutoff	Changed proteins	Reference
11/06/2011	Mouse T-cell hybridoma cells	0 h vs 4 h chase with palmitic acid	MLCC (17-ODYA)	SILAC2plex	>2.0	~80	[27]
11/06/2011	Mouse T-cell hybridoma cells	4 h HDFPa) vs DMSO	MLCC (17-ODYA)	SILAC2plex	>1.5	~50	[27]
08/16/2012	Parasite *Plasmodium falciparum*	6 h 2BP vs DMSO	ABE	Stable isotope dimethyl labeling	<0.782	67	[31]
11/07/2013	Mouse brains	WT vs YAC128	ABE	SILAM	>1.1	19	[34]
12/19/2013	Human umbilical vein endothelial cells	6 h insulin vs vehicle	ABE	SILAC2plex	>+2.0 or <−3.0	26	[35]

a) hexadecylfluorophosphonate

## Abbreviations

17-ODYA: 17-Octadecynoic Acid
2BP: 2-Bromopalmitate
2BPN3: ω-Azido Analog of 2-Bromopalmitate
ABE: Acyl-Biotinyl Exchange
APT: Acylprotein Thioesterase
cICAT: Cleavable Isotope-Coded Affinity Tag
CKAP4: Cytoskeleton-Associated Protein 4
DHHC: Asp-His-His-Cys
FDR: False Discovery Rate
GT: Gene Trapped
HDYOA: 15-Hexadecynyloxyacetic Acid
HIP14: Huntingtin-Interacting Protein 14
iTRAQ: Isobaric Tagging for Relative and Absolute Quantification
MLCC: Metabolic Labeling with a Palmitic Acid Analog followed by Click Chemistry
MS: Mass Spectrometry
PalmPISC: Palmitoyl Protein Identification and Site Characterization
PAT: Palmitoyl Acyltransferase
PICA: Palmitoyl- Cysteine Isolation Capture and Analysis
RAC: Resin-Assisted Capture
SC: Spectral Counting
SILAC: Stable Isotope Labeling by Amino Acids in Cell Culture
SILAM: Stable Isotope Labeling in Mammals
TCEP: Tris(2-Carboxyethyl)Phosphine
TIP1: Tip Growth Defective 1
TPC: Thiopropyl Captivation
